# Limitations of global end-diastolic volume index as a parameter of cardiac preload in the early phase of severe sepsis: a subgroup analysis of a multicenter, prospective observational study

**DOI:** 10.1186/2052-0492-1-11

**Published:** 2013-11-28

**Authors:** Tomoyuki Endo, Shigeki Kushimoto, Satoshi Yamanouchi, Teruo Sakamoto, Hiroyasu Ishikura, Yasuhide Kitazawa, Yasuhiko Taira, Kazuo Okuchi, Takashi Tagami, Akihiro Watanabe, Junko Yamaguchi, Kazuhide Yoshikawa, Manabu Sugita, Yoichi Kase, Takashi Kanemura, Hiroyuki Takahashi, Yuuichi Kuroki, Hiroo Izumino, Hiroshi Rinka, Ryutarou Seo, Makoto Takatori, Tadashi Kaneko, Toshiaki Nakamura, Takayuki Irahara, Nobuyuki Saito, PiCCO Pulmonary Edema Study Group

**Affiliations:** Department of Emergency and Critical Care Medicine, Tohoku University Hospital, Sendai, Miyagi, 980-8574 Japan; Division of Emergency Medicine, Tohoku University Graduate School of Medicine, Sendai, Miyagi, 980-8574 Japan; Department of Emergency and Critical Care Medicine, Kurume University School of Medicine, Kurume-shi, Fukuoka, 830-0011 Japan; Department of Emergency and Critical Care Medicine, Faculty of Medicine, Fukuoka University, Fukuoka, Fukuoka, 814-0180 Japan; Department of Emergency and Critical Care Medicine, Kansai Medical University, Moriguchi, Osaka, 570-8506 Japan; Department of Emergency and Critical Care Medicine, St. Marianna University School of Medicine, Kawasaki, Kanagawa, 216-8511 Japan; Department of Emergency and Critical Care Medicine, Nara Medical University, Kashihara, Nara, 634-8521 Japan; Department of Emergency and Critical Care Medicine, Nippon Medical School Hospital, Bunkyo-ku, Tokyo, 113-8603 Japan; Department of Emergency and Critical Care Medicine, Nihon University School of Medicine Itabashi Hospital, Itabashi-ku, Tokyo, 173-8610 Japan; Shock Trauma and Emergency Medical Center, Tokyo Medical and Dental University Hospital of Medicine, Bunkyo-ku, Tokyo, 113-8519 Japan; Department of Emergency and Critical Care Medicine, Juntendo University Nerima Hospital, Nerima-ku, Tokyo, 177-8521 Japan; Department of Critical Care Medicine, Jikei University School of Medicine, Minato-ku, Tokyo, 105-8471 Japan; Emergency and Critical Care Medicine, National Hospital Organization Disaster Medical Center, Tachikawa-shi, Tokyo, 190-0014 Japan; Department of Intensive Care Medicine, Saiseikai Yokohamashi Tobu Hospital, Kanagawa, Japan; Department of Emergency and Critical Care Medicine, Social Insurance Chukyo Hospital, Nagoya, Aichi, 457-8510 Japan; Advanced Emergency and Critical Care Center, Kansai Medical University Takii Hospital, Moriguchi, Osaka, 570-8507 Japan; Emergency and Critical Care Medical Center, Osaka City General Hospital, Miyakojima, Osaka, 534-0021 Japan; Intensive Care Unit, Kobe City Medical Center General Hospital, Kobe, Hyogo, 650-0046 Japan; Department of Anesthesia and Intensive Care, Hiroshima City Hospital, Hiroshima-shi, Hiroshima, 730-8518 Japan; Advanced Medical Emergency and Critical Care Center, Yamaguchi University Hospital, Ube, Yamaguchi, 755-8505 Japan; Intensive Care Unit, Nagasaki University Hospital, Sakamoto, Nagasaki, 852-8501 Japan; Department of Emergency and Critical Care Medicine, Nippon Medical School Tama Nagayama Hospital, Tama-shi, Tokyo, 206-8512 Japan; Department of Emergency and Critical Care Medicine, Nippon Medical School Chiba Hokusou Hospital, Inzai-shi, Chiba, 270-1694 Japan

**Keywords:** Sepsis-induced myocardial dysfunction, Global end-diastolic volume index, Stroke volume variation, Diastolic dysfunction, Severe sepsis

## Abstract

**Background:**

In patients with severe sepsis, depression of cardiac performance is common and is often associated with left ventricular (LV) dilatation to maintain stroke volume. Although it is essential to optimize cardiac preload to maintain tissue perfusion in patients with severe sepsis, the optimal preload remains unknown. This study aimed to evaluate the reliability of global end-diastolic volume index (GEDI) as a parameter of cardiac preload in the early phase of severe sepsis.

**Methods:**

Ninety-three mechanically ventilated patients with acute lung injury/acute respiratory distress syndrome secondary to sepsis were enrolled for subgroup analysis in a multicenter, prospective, observational study. Patients were divided into two groups—with sepsis-induced myocardial dysfunction (SIMD) and without SIMD (non-SIMD)—according to a threshold LV ejection fraction (LVEF) of 50% on the day of enrollment. Both groups were further subdivided according to a threshold stroke volume variation (SVV) of 13% as a parameter of fluid responsiveness.

**Results:**

On the day of enrollment, there was a positive correlation (*r* = 0.421, *p* = 0.045) between GEDI and SVV in the SIMD group, whereas this paradoxical correlation was not found in the non-SIMD group and both groups on day 2. To evaluate the relationship between attainment of cardiac preload optimization and GEDI value, GEDI with SVV ≤13% and SVV >13% was compared in both the SIMD and non-SIMD groups. SVV ≤13% implies the attainment of cardiac preload optimization. Among patients with SIMD, GEDI was higher in patients with SVV >13% than in patients with SVV ≤13% on the day of enrollment (872 [785–996] mL/m^2^ vs. 640 [597–696] mL/m^2^; *p* < 0.001); this finding differed from the generally recognized relationship between GEDI and SVV. However, GEDI was not significantly different between patients with SVV ≤13% and SVV >13% in the non-SIMD group on the day of enrollment and both groups on day 2.

**Conclusions:**

In the early phase of severe sepsis in mechanically ventilated patients, there was no constant relationship between GEDI and fluid reserve responsiveness, irrespective of the presence of SIMD. GEDI should be used as a cardiac preload parameter with awareness of its limitations.

## Background

In patients with severe sepsis, depression of cardiac performance has been described for more than three decades. Such sepsis-induced myocardial dysfunction (SIMD) is acute, is reversible, and has a high incidence of approximately 40% in patients with severe sepsis [1–5].

Although the relationship between SIMD and left ventricular (LV) dilatation has been demonstrated, the effect of compensatory LV dilatation is controversial in the clinical setting. Several studies have reported that LV dilatation, which means increased LV compliance, might be a protective mechanism associated with better survival in patients with reduced LV ejection fraction (LVEF) [1, 5–7]. In contrast, a study indicated that such LV dysfunction was related to poor prognosis [8]. In addition to LV dilatation, the right ventricle has also been shown to be dilated because of right ventricular dysfunction in SIMD [1, 3, 4, 9, 10]. Therefore, global end-diastolic volume would be increased by biventricular dysfunction in SIMD.

Although it is essential to optimize cardiac preload immediately to maintain tissue perfusion in patients with severe sepsis and signs of tissue hypoperfusion, it is difficult to appropriately evaluate the status of cardiac preload in patients with SIMD during the early phase of severe sepsis [11]; moreover, a reliable parameter for cardiac preload has not been elucidated thus far. Recently, the transpulmonary thermodilution (TD) system has been widely used in the critical care setting. Global end-diastolic volume index (GEDI), a static volumetric parameter in TD, is considered more eligible for determining cardiac preload than central venous pressure or pulmonary artery wedge pressure in patients with septic shock [12]. Despite the usefulness of GEDI as a preload parameter, the optimal value of GEDI in patients with SIMD has not been investigated and is expected to be increased compared to that in sepsis patients with preserved cardiac function. We hypothesized that the optimal GEDI, as the preload parameter, would be greater in patients with SIMD than in those without SIMD. In the present study, we aimed to evaluate the difference between GEDI in patients with and without SIMD and to determine the reliability of GEDI as a parameter of cardiac preload during the early phase of severe sepsis.

## Methods

This is a subgroup analysis of a multicenter, prospective, observational study conducted to clarify the clinical features of acute respiratory distress syndrome (ARDS) and to establish quantitative diagnostic criteria [13]. The detailed study protocol has been described in the original article. The study was approved by the ethics committee of each of the 23 institutions, and written informed consent was provided by each patient’s next of kin. The investigation was registered with the University Hospital Medical Information Network (UMIN) Clinical Trials Registry, UMIN-CTR ID UMIN000003627. Between March 2009 and August 2011, 301 patients were enrolled in this study. The inclusion criteria were age ≥15 years, requirement of mechanical ventilation (expected >48 h) for acute respiratory failure with a partial pressure of arterial oxygen (PaO_2_)/fraction of inspired oxygen (FiO_2_) ratio of ≤300 mmHg, and bilateral infiltration on chest radiography. Patients with a history of pulmonary resection, pulmonary thromboembolism, severe peripheral arterial disease, a cardiac index of <1.5 L/min/m^2^, lung contusion, burns, and other conditions unsuitable for evaluation with the TD technique were excluded. Data sampling was performed once a day for 3 days. Among the 301 patients, 207 were considered to have acute lung injury (ALI)/ARDS. Ninety-three patients with ALI/ARDS secondary to sepsis whose LVEF was evaluated by transthoracic echocardiography on the day of enrollment were enrolled for this subgroup analysis. Because the method to measure LVEF was not predetermined, each institution had selected either the single-plane area-length method or modified Simpson method according to the presence of LV asynergy. Depending on the human resources of each institution, a registered medical sonographer, a cardiologist, an intensivist, or an emergency physician performed the LVEF measurement. Patients were divided into two groups according to LVEF on the day of enrollment. In this study, the threshold value of LVEF with SIMD was defined as 50%, as previously described [3, 8, 14], which was sufficiently lower than the normal echocardiographic LVEF of 66% ± 5% (SD) in the Japanese population [15]. To investigate the fluid reserve responsiveness in these patients, both groups were further subdivided into two subgroups according to stroke volume variation (SVV), and SVV threshold was defined as 13%, as previously described [16, 17]. In mechanically ventilated patients, SVV is a useful predictor of fluid responsiveness and has better sensitivity and specificity than GEDI [18–24]. SVV ≤13% indicates low fluid reserve responsiveness and the attainment of cardiac preload optimization.

### Measurement of GEDI, EVLWi, PVPI, and SVV

A thermistor-tipped catheter was connected to the PiCCO® plus or PiCCO® 2 monitor (Pulsion Medical Systems, Munich, Germany). This monitor uses a single-thermal indicator technique to calculate the cardiac output, global end-diastolic volume (GEDV), and extravascular lung water (EVLW). GEDV is calculated as the difference between the intrathoracic thermal volume and pulmonary thermal volume, which represents the combined end-diastolic volumes of the four cardiac chambers. Intrathoracic blood volume was calculated as 1.25 × GEDV − 28.4 [25]. EVLW is the difference between the intrathoracic thermal volume and intrathoracic blood volume [25]. Pulmonary vascular permeability index (PVPI) was calculated as the ratio of EVLW to pulmonary blood volume [26]. The absolute EVLW and GEDV values were indexed to predicted body weight [27–29] and provided as extravascular lung water index (EVLWi) and GEDI, respectively. The PiCCO® plus or PiCCO® 2 monitor can also automatically calculate the SVV, which is a dynamic parameter of fluid responsiveness [18–24]. We recorded the SVV value at the time of GEDI measurement.

### Statistical analysis

Data are presented as median (interquartile range (IQR)). The proportions were compared using Pearson’s chi-square test or Fisher’s exact test, as required. Spearman’s rank correlation coefficient was used for determining the correlation between variables, and Mann-Whitney’s *U* test was used for assessing the differences between groups. A *p* value <0.05 was considered significant. All statistical analyses were performed using SPSS 19.0 for Windows (SPSS, Chicago, IL, USA).

## Results

### Characteristics of patients with ALI/ARDS secondary to sepsis on the day of enrollment

Table 1 shows the patient characteristics on the day of enrollment. Ninety-three patients were evaluated for inclusion in the current analysis. Of those, 23 patients were diagnosed with SIMD, which was defined as LVEF ≤50%.

**Table 1:** **Characteristics of patients with ALI/ARDS secondary to sepsis on the day of enrollment**

Characteristics	SIMD group	Non-SIMD group	***p*** value
	***n*** = 23	***n*** = 70	
Age, years	79 (64–83)	72 (62–82)	0.430
Gender (male), *n* (%)	15 (65.2)	42 (60.0)	0.806
Septic shock, *n* (%)	14 (60.9)	40 (57.1)	0.899
Vasoactive-inotropic agents, *n* (%)	21 (91.3)	50 (71.4)	0.087
APACHE II score, points	24 (19–27)	25 (19–30)	0.565
SOFA score, points	12 (10–14)	11 (9–13)	0.345
PaO_2_/FIO_2_ ratio, mmHg	128 (80–198)	134 (96–185)	0.943
GEDI, mL/m^2^	757 (644–892)	837 (669–934)	0.293
EVLWi, mL/kg	16.2 (13.2–21.5)	18.2 (14.2–23.6)	0.287
PVPI	2.9 (2.4–3.7)	3.1 (2.4–3.9)	0.599
28-day mortality, *n* (%)	9 (39.1)	32 (45.7)	0.635

All data are presented as median (interquartile range) unless otherwise stated. *ALI/ARDS* acute lung injury/acute respiratory distress syndrome, *APACHE* Acute Physiology and Chronic Health Evaluation, *SIMD* sepsis-induced myocardial dysfunction, *SOFA* Sequential Organ Failure Assessment, *GEDI* global end-diastolic volume index, *EVLWi* extravascular lung water index, *PVPI* pulmonary vascular permeability index.

There were no significant differences in age, gender, prevalence of septic shock, APACHE II score, SOFA score, PaO_2_/FiO_2_ ratio, GEDI, EVLWi, PVPI, and 28-day mortality between patients with SIMD and without SIMD (non-SIMD).

### Relationship between GEDI, SVV, and LVEF

**Figure 1:**
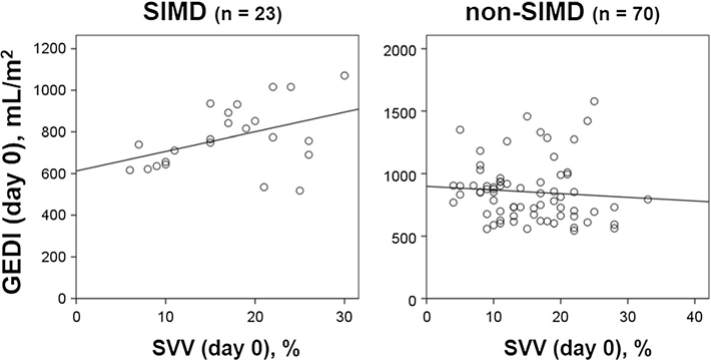
**Relationship between SVV and GEDI on the day of enrollment (day 0).** There was a moderate positive correlation (*r* = 0.421, *p* = 0.045) between GEDI and SVV in the SIMD group, whereas no such correlation was noted in the non-SIMD group (*r* = −0.081, *p* = 0.503). SIMD, sepsis-induced myocardial dysfunction; GEDI, global end-diastolic volume index; SVV, stroke volume variation.

**Figure 2:**
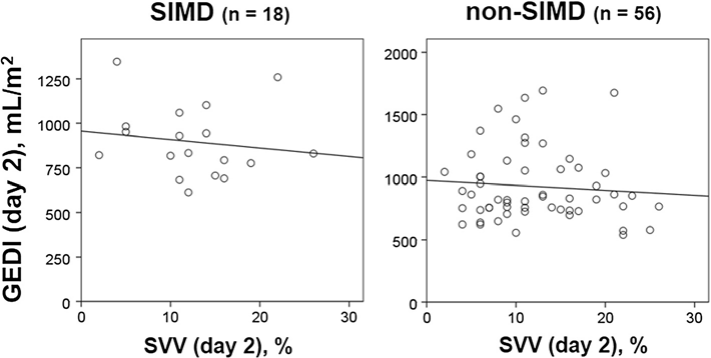
**Relationship between SVV and GEDI on day 2.** There were no correlations between GEDI and SVV in both groups (SIMD group: *r* = −0.148, *p* = 0.557; non-SIMD group: *r* = −0.083, *p* = 0.545). SIMD, sepsis-induced myocardial dysfunction; GEDI, global end-diastolic volume index; SVV, stroke volume variation.

Initially, we analyzed the relationship between GEDI and SVV in both the SIMD and non-SIMD groups (Figures 1 and 2). On the day of enrollment (Figure 1), there was a moderate positive correlation (*r* = 0.421, *p* = 0.045) between GEDI and SVV in the SIMD group, whereas this correlation was not found in the non-SIMD group (*r* = −0.081, *p* = 0.503). On day 2 (Figure 2), there were no correlations between GEDI and SVV in both groups (SIMD, *r* = −0.148, *p* = 0.557; non-SIMD, *r* = −0.083, *p* = 0.545).

**Figure 3:**
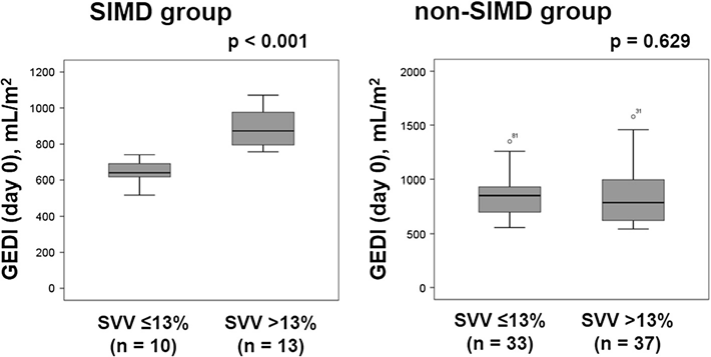
**Comparison of GEDI between patients with SVV ≤13% and SVV >13% on day 0.** Among patients with the SIMD group, GEDI was significantly higher in patients with SVV >13% than in patients with SVV ≤13% on the day of enrollment (SVV >13%, 872 [785–996] mL/m^2^; SVV ≤13%, 640 [597–696] mL/m^2^; *p* < 0.001). In contrast, GEDI was not significantly different between patients with SVV ≤13% and SVV >13% in the non-SIMD group on the day of enrollment (SVV ≤13%, 852 [687–934] mL/m^2^; SVV >13%, 787 [620–998] mL/m^2^; *p* = 0.629). SIMD, sepsis-induced myocardial dysfunction; GEDI, global end-diastolic volume index; SVV, stroke volume variation.

**Figure 4:**
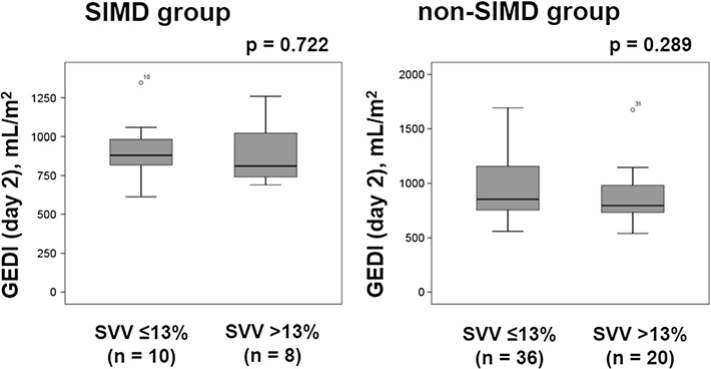
**Comparison of GEDI between patients with SVV ≤13% and SVV >13% on day 2.** On day 2, in both the SIMD and non-SIMD groups, there were no significant differences in the levels of GEDI between patients with SVV ≤13% and SVV >13% (SIMD with SVV ≤13%, 881 [784–1,001] mL/m^2^ vs. SIMD with SVV >13%, 811 [724–1,062] mL/m^2^, *p* = 0.722; non-SIMD with SVV ≤13%, 853 [754–1,170] mL/m^2^ vs. non-SIMD with SVV >13%, 795 [730–1,007] mL/m^2^, *p* = 0.289). SIMD, sepsis-induced myocardial dysfunction; GEDI, global end-diastolic volume index; SVV, stroke volume variation.

To evaluate the relationship between attainment of cardiac preload optimization and GEDI value, we compared GEDI between patients with SVV ≤13% and SVV >13% in both the SIMD and non-SIMD groups (Figures 3 and 4). Among the patients with SIMD, GEDI was significantly higher in those with SVV >13% than in those with SVV ≤13% on the day of enrollment (SVV >13%, 872 [785–996] mL/m^2^; SVV ≤13%, 640 [597–696] mL/m^2^; *p* < 0.001). In contrast, GEDI was not significantly different between patients with SVV ≤13% and SVV >13% in the non-SIMD group on the day of enrollment (SVV ≤13%, 852 [687–934] mL/m^2^; SVV >13%, 787 [620–998] mL/m^2^; *p* = 0.629) (Figure 3). On day 2, in both the SIMD and non-SIMD groups, there were no significant differences in the levels of GEDI between patients with SVV ≤13% and SVV >13% (SIMD with SVV ≤13%, 881 [784–1001] mL/m^2^ vs. SIMD with SVV >13%, 811 [724–1062] mL/m^2^, *p* = 0.722; non-SIMD with SVV ≤13%, 853 [754–1170] mL/m^2^ vs. non-SIMD with SVV >13%, 795 [730–1007] mL/m^2^, *p* = 0.289) (Figure 4).

## Discussion

Parker et al. published a landmark article in the early 1980s demonstrating SIMD [5]. They suggested that adequate LV stroke output can be maintained through acute compensatory LV dilatation despite a severely depressed LVEF, which was calculated using radionuclide cineangiography. The methods used for evaluating cardiac preload and LVEF in sepsis patients have changed from cineangiography to pulmonary artery catheterization, echocardiography, and TD; these methods can be used to obtain accurate LV volume measurements at the bedside [30]. Although Parker et al. demonstrated 100% LV dilatation (from 100 to 200 mL) in patients with systolic dysfunction using cineangiography [5], other investigators have reported that the rate of increase in LV end-diastolic volume is less than 20% using echocardiography [3, 6]. These discordant findings may be because both the right and LV chambers are enclosed in the relatively stiff pericardium that restricts expansion of the entire heart. For such compensatory dilated heart, the fluid reserve responsiveness and the optimal GEDI value that does not cause left atrial hypertension are still unknown. Dynamic hemodynamic parameters such as SVV and pulse pressure variation (PPV) are now available; these parameters indicate the part of the Frank-Starling curve in which the cardiac preload is located [24]. If SVV/PPV shows ≤13% variation, there is low fluid reserve responsiveness, and the cardiac preload may be located on the plateau part of the Frank-Starling curve [16, 17]. Therefore, by evaluating GEDI and SVV simultaneously, we could estimate whether the measured GEDI was located on the plateau part of the Frank-Starling curve. Considering the abovementioned findings, in this study, we analyzed the relationship between LVEF, GEDI, and SVV in order to evaluate the reliability of GEDI as a parameter of cardiac preload in the early phase of severe sepsis.

In an intensive care unit setting, it is necessary to consider extracardiac factors such as positive airway pressure ventilation, pulmonary hypertension secondary to lung injury, and afterload alteration by vasopressors; hence, the proposed GEDI reference range may not be suitable for such critically ill patients. Recently, Eichhorn et al. analyzed the published data for GEDI between a cohort of septic patients and patients undergoing major surgery [31]. In their analysis, the pooled estimate for the mean value for GEDI was 788 mL/m^2^ in septic patients and 694 mL/m^2^ in surgical patients; this finding suggested that the mean GEDI was significantly higher in sepsis patients than in the surgical group. Despite this finding, the optimal GEDI value in patients with sepsis and those with SIMD is still unknown.

At present, several methods are available to evaluate myocardial dysfunction, such as cineangiography, cinescintigraphy, magnetic resonance imaging, cardiac index measured by TD, and echocardiography. Among those methods, both cardiac index by TD and echocardiography are useful for the bedside evaluation of mechanically ventilated patients. Although cardiac index can be measured continuously by the TD system as a hemodynamic parameter, it could be affected by alteration of heart rate, preload, afterload, right ventricular function, and LV compliance. Moreover, cardiac index would not necessarily be depressed in sepsis with SIMD [1, 3, 8]. We considered that echocardiography would be more eligible for this study as it had been widely used in previous studies to evaluate the presence of SIMD and could directly visualize the cardiac performance [1, 3, 4, 6–8, 10, 14]. Therefore, we defined SIMD by LVEF measured by transthoracic echocardiography. The threshold value of LVEF for SIMD was defined as 50% based on the previous studies [3, 8, 14].

In our results, there was no significant difference in GEDI between patients with SIMD and non-SIMD on the day of enrollment. Although the extent of fluid resuscitation was not known, these GEDI values were similar to the results reported by Eichhorn et al. [31]. Contrary to our expectation, the GEDI in patients with SIMD was not necessarily greater than that in patients without SIMD during early sepsis.

Analysis of the relationship between GEDI and SVV showed that the correlation was paradoxical in the SIMD group on the day of enrollment. In the second analysis, we found that on the day of enrollment, the GEDI in patients with SVV ≤13% was significantly lower than that in patients with SVV >13% in the SIMD group. These findings are different from the generally recognized relationship between GEDI and SVV; this implies that greater GEDI is usually associated with lower SVV according to adequate cardiac filling. Although several interpretations for this paradoxical relationship may be considered—such as a limitation of the static parameters (GEDI) to predict fluid responsiveness compared with the dynamic parameters (SVV) [19, 20], a large interindividual variance of GEDI [32], the racial difference that the Japanese have smaller hearts than non-Asians [15], and the presence of LV diastolic dysfunction that impairs compensatory LV enlargement [4]—none of these explanations are definitive.

In contrast, there was no significant difference between GEDI in patients with SVV ≤13% and SVV >13% among the non-SIMD group on the day of enrollment, and the same results were found between both SVV groups on day 2, regardless of the LVEF. These findings are also different from the generally recognized relationship between GEDI and SVV mentioned above. Since we were unable to identify a definite relationship between GEDI and SVV in patients with or without SIMD, the optimal GEDI value for these patients could not be defined in the present study.

### Limitations

This subgroup analysis has several limitations. This is a retrospective analysis with a small sample size. Since the original study excluded patients with more than 5 days from the onset of acute respiratory failure, the exact date of occurrence of sepsis in each patient was unknown [13]. The ventilator settings, such as tidal volume (reliable when tidal volume is at least 8 mg/kg), spontaneous mode (pressure support ventilation), and airway pressure release ventilation mode, may have influenced SVV measurement [33, 34]. In this study, the ventilator mode depended on each institution’s policy; therefore, SVV as a predictor of fluid responsiveness was not an adequate measurement for some patients. Although the use of muscle relaxants may affect the SVV, we did not examine their use in the original study and could not evaluate their influence on SVV. In the protocol of the present study, the method used to measure LVEF by transthoracic echocardiography was not uniform. There was a possibility that LVEF in some patients with SIMD may have improved on day 2 and such an improvement could have affected the results of the present study.

## Conclusions

During the early phase of patients with severe sepsis on mechanical ventilation, there was no constant relationship between GEDI and fluid reserve responsiveness, irrespective of the presence of SIMD, defined as LVEF ≤50%. Since the absolute value of optimal GEDI, indicating adequate cardiac filling, may not be determined regardless of the presence of SIMD, GEDI should be used as a cardiac preload parameter with awareness of its limitations.

## Abbreviations

ALI: Acute lung injury
ARDS: Acute respiratory distress syndrome
EVLW: Extravascular lung water
EVLWi: Extravascular lung water index
GEDI: Global end-diastolic volume index
GEDV: Global end-diastolic volume
LV: Left ventricular
LVEF: Left ventricular ejection fraction
PPV: Pulse pressure variation
PVPI: Pulmonary vascular permeability index
SIMD: Sepsis-induced myocardial dysfunction
SVV: Stroke volume variation
TD: Thermodilution.
